# Nursing Roles in the Quality of Information in Informed Consent Forms of a Spanish County Hospital

**DOI:** 10.3390/nursrep14010008

**Published:** 2024-01-04

**Authors:** José Manuel García-Álvarez, Alfonso García-Sánchez

**Affiliations:** 1 Health Sciences Program, Catholic University of Murcia (UCAM), Guadalupe, 30107 Murcia, Spain; 2 Faculty of Nursing, Catholic University of Murcia (UCAM), Guadalupe, 30107 Murcia, Spain; agarcia@ucam.edu

**Keywords:** informed consent forms, readability, comprehension, formal quality, nursing professionals

## Abstract

(1) Background: Because of their direct and continuous contact with the patient, nurses play a relevant role in ensuring that informed consent forms are complete and easy to read and comprehend. The objective of this study was to analyze the legibility and formal quality of informed consent forms for non-surgical procedures in a county hospital. (2) Methods: The readability of these forms was analyzed using the INFLESZ scale and the information they provided according to the formal quality criteria established for these forms. (3) Results: Readability was difficult in 78.08% of the forms analyzed. No form fulfilled all the criteria, the most non-compliant being the non-appearance of the verification of delivery of a copy to the patient (100%), the contraindications (94.59%), and the alternatives (83.78%) of the procedure. Statistically significant differences were observed between disciplines with respect to the INFLESZ readability score and the formal quality score, but no statistically significant correlation was found between the two scores. (4) Conclusions: The informed consent forms for non-surgical procedures analyzed presented mostly difficult readability and poor formal quality, making it difficult for patients to have understandable and complete information. Nursing professionals should be actively involved in their improvement to facilitate patient decision making.

## 1. Introduction

The current technological development of health sciences has created the need to establish a new nexus to link the scientific, legal, and ethical aspects involved in the relationship established between patients and health professionals [1].

This important task of linking these three aspects is carried out by the informed consent process related to the procedure the patient is going to undergo. This process is responsible for legally and ethically guaranteeing patients’ freedom of choice in the face of any health procedure, allowing patients to participate voluntarily and actively in all decisions that affect their health in any way [2]. Ethical and legal regulations also aim to make decision making transparent and fair. The patient should be able to make a decision based on the technical and scientific information provided by the health professional [3]. To achieve this patient empowerment, it is necessary that the information they receive about the procedure to be performed is clear, sufficient, in accordance with current scientific knowledge, and appropriate to the personal characteristics of each patient. In this way, an adequate comprehension of the information transmitted is favored, and the patient’s decision-making process is facilitated [4,5].

Once patients have been informed about the procedure they are going to undergo, they must give their authorization for it to be performed. Although both the information and the authorization can be given orally, it is preferable that both be recorded in writing using a specific form for each procedure. The informed consent form is required for procedures that are very complex or that may cause significant health risks to patients [6,7,8,9].

The informed consent form is of great importance in the relationship established between patients and healthcare professionals, as it serves to transmit the information that the patient needs to decide and accept the specific procedure he is going to undergo. It is therefore essential that the informed consent form is properly drafted so that the information it contains can be fully comprehended by the specific patient to whom it is addressed. In addition, it must contain all the relevant information that the patient may need to make a decision that meets his or her needs and preferences [10,11,12,13].

Any informed consent form should present a structure and language appropriate to the specific characteristics of the patients to whom it may be addressed in order to facilitate its reading and improve its comprehension [10,14].

The lack of information or the difficulty in understanding it means that the informed consent form ceases to be an effective tool and becomes a mere bureaucratic formality aimed at avoiding any legal liability [15]. Although it is not always possible to achieve these objectives, especially in patients with serious pathologies in whom treatments without sufficient scientific evidence are used as a last resort [16].

Readability is a quality that makes it possible to objectively evaluate the ease of reading of written documents by means of mathematical formulas that mainly measure the length of sentences and words. It is thought that the shorter the sentences and words used in the informed consent form, the easier it should be for patients to read and comprehend. Readability allows these forms to adequately fulfill their informative function. There are different tools to measure the readability of a document, such as the Flesch–Kincaid Reading Index, the Fernandez-Huerta Index, the Flesch–Szigriszt Index, and the INFLESZ scale [10,14].

Studies carried out with patients to analyze the readability of different healthcare documents, including informed consent forms, indicate that they tend to have a low readability that makes them difficult to comprehend. This reading difficulty means that, in general, informed consent forms need to be improved [10,14].

It is necessary to ensure that the informed consent forms are of good formal quality and contain all the information that patients need to be able to decide in a way that is appropriate to their needs and interests. For this purpose, these forms should include the following information concerning the procedure to which the patient is going to be submitted: description, purpose, benefits, relevant consequences, typical risks, contraindications, alternatives, and personalized risks. In addition, to comply with legal requirements, informed consent forms are obliged to include the date of issue, hospital identification data, the identification data and signatures of the healthcare professional and the patient, and a section allowing revocation at any time [11,17,18,19]. The lack or failure to complete any of these sections legally and ethically invalidates the informed consent form by not providing patients with the necessary information to enable them to make their decision freely and voluntarily [12,20,21].

Nursing professionals have an important role in the informed consent process as they carry out their healthcare work in direct and continuous contact with patients and their families. Therefore, nursing professionals are also responsible for ensuring that patients are adequately informed about the procedures they are going to undergo. Nursing professionals must ensure the patient’s autonomy at all times by means of a helping relationship focused on encouraging their voluntary and active participation in everything that affects their health. These healthcare professionals should also be responsible for evaluating the patient’s degree of comprehension of the informed consent form. In addition, as they frequently accompany the patient during its reading, they can answer any questions or doubts that may arise due to the biomedical language used in these forms. Therefore, the nursing professional has a very relevant role in facilitating this process, and specifically the development and review of the informed consent form, to truly fulfill the legal and ethical function entrusted to it [22,23,24].

The objective of the present study was to analyze the readability and formal quality of the informed consent forms for non-surgical procedures in a county hospital in Spain and, if necessary, to make the appropriate modifications to ensure that these forms adequately fulfill their function.

## 2. Materials and Methods

An observational cross-sectional study was conducted to analyze the readability and formal quality of the informed consent forms for non-surgical procedures at the “Vega Lorenzo Guirao” Hospital (Cieza, Spain) in March 2023.

This study analyzed the informed consents of those disciplines that usually perform their interventional procedures outside the surgical area. The non-surgical disciplines analyzed were allergy and immunology, cardiology, gastroenterology, hematology, pulmonology, neurology, and radiology.

The INFLESZ scale based on the Flesch–Szigriszt Index was used to assess the readability of informed consent forms for non-surgical procedures. This scale is specific to the reading habits of the Spanish population and has been validated to analyze the readability of Spanish healthcare texts. This scale evaluates the readability of healthcare documents addressed to patients, establishing a score according to the length of sentences and words by using the following mathematical formula: 206.835 − 62.35 × (total syllables/total words) − (total words/total sentences). The INFLESZ scale establishes five degrees of reading ease that correspond to the level of education that readers must have in order to be able to read and comprehend a given type of publication (Table 1). This scale establishes as normal the readability score obtained by adult publications in newsstands because their reading is accessible to the average Spanish citizen. A readability score equal to or higher than 55 indicates a greater probability of being comprehended by patients [25].

The formal quality of the hospital’s informed consent forms for non-surgical procedures was assessed using the formal quality criteria established by different studies carried out in Spain [11,17] (Table 2). We analyzed whether or not the forms complied with these criteria, assigning one point to all affirmative responses and zero points to negative responses, to finally obtain a formal quality score for each of the forms analyzed.

The statistical program IBM SPSS Statistics for Windows (Version 25.0. Armonk, NY, USA: IBM Corp.) was used to analyze the information obtained in this study. Frequencies and percentages were calculated for qualitative variables, as well as the mean and standard deviation for quantitative variables. To evaluate the associations between the different variables present in this study, ANOVA, Chi-square, Kruskal–Wallis and Spearman tests were used according to the characteristics of each of the variables, using a *p* value < 0.05 to indicate significance. Analysis of variance (ANOVA) was used to analyze the association between the type of specialty (a nominal qualitative polytomous variable) and the INFLESZ readability score (a continuous quantitative variable). The Chi-square test was used to analyze the association between type of specialty and formal quality criteria (polytomous nominal qualitative variable). The Kruskal–Wallis test was used to analyze the association between type of specialty and formal quality score (ordinal quantitative variable). Spearman’s correlation coefficient was used to determine whether there is a relationship between the INFLESZ readability score and the formal quality score.

We used the STROBE guidelines to ensure adequate reporting of our findings [26].

## 3. Results

Table 3 shows the INFLESZ readability score and the formal quality score obtained by the 37 informed consent forms for different procedures in the seven non-surgical disciplines of the hospital.

The mean readability score of the informed consent forms for non-surgical procedures analyzed according to the INFLESZ scale was 50.76, with a standard deviation of 5.60. Twenty-eight forms (75.68%) presented a “somewhat difficult” degree of readability (score between 40 and 55), eight forms (21.62%) obtained a “normal” degree of readability (score between 55 and 65), and one form (2.70%) presented a “very difficult” degree of readability (score less than 40) (Table 4). The discipline of allergy and immunology, with a single form, presented the greatest reading difficulty with a score of 42.65, while the discipline of pulmonology, with nine forms, obtained the greatest ease of reading with a mean score of 54.42 (Table 3).

The informed consent forms for non-surgical procedures analyzed had a minimum non-compliance with one criterion and a maximum of six formal quality criteria (Table 4). None of the disciplines met all the criteria analyzed. The most non-compliant formal quality criteria were the verification of the delivery of a copy to the patient (item 19) in 100% of the forms, the contraindications of the procedure (item 13) in 94.59% of the forms, the alternatives to the procedure (item 14) in 83.78% of the forms, and the personalized risks of the procedure (item 12) in 5.40% of the forms (Table 5).

The informed consent forms for non-surgical procedures analyzed presented a formal quality score with a maximum of 18 points and a minimum of 13 points (Table 4) and a mean score of 16.08 (Table 3). Of the formal quality scores obtained after analyzing these forms, 72.97% corresponded to the value of 16 points (27 forms) and 16.22% to the value of 17 points (6 forms) (Table 4).

Table 6 describes the inferential statistical analysis performed between the different variables, indicating the statistical test used and the statistical significance obtained. Statistically significant associations were observed between informed consent forms across disciplines with the INFLESZ readability score, compliance with formal quality criteria, and the formal quality score. No statistically significant correlation was observed between the INFLESZ readability score and the formal quality score.

## 4. Discussion

The informed consent forms for non-surgical procedures analyzed in this study mostly presented a degree of readability according to the INFLESZ scale of “somewhat difficult” (score between 40 and 55). The complexity in the wording of these forms makes it difficult for patients to achieve sufficient comprehension to make decisions according to their needs and interests [28,29].

In this study, the INFLESZ scale was chosen to assess the readability of non-surgical informed consent forms, as it is a specific and validated tool for its analysis in Spanish healthcare texts [25]. Although similar results are observed in other studies that have used other scales to evaluate the readability of informed consent forms [30,31,32,33,34]. Therefore, regardless of the tool used to assess legibility, these forms presented reading difficulties for a significant proportion of patients that would prevent adequate comprehension.

The information contained in the informed consent forms for non-surgical procedures analyzed would be equivalent to that presented in specialized newspapers in different fields or in articles used for scientific dissemination. These texts are difficult to read, and therefore to comprehend, by a large majority of the population [25]. The readability of these forms would need to be improved so that they have an INFLESZ readability score of 55 or higher and can be easily read and comprehended by the majority of patients. Some of the recommendations that have proven useful for improving the readability of these forms are: try to limit these forms to one page; try to keep most of the sentences they contain short and simple; place the most relevant information at the beginning of each paragraph to capture the patient’s attention more easily; try to avoid the use of very specific health terms or acronyms without their corresponding explanation; increase the spaces between paragraphs; use different fonts to highlight the most relevant information. Another very useful option to improve the readability of these forms would be their adaptation by a person outside the healthcare profession who is properly trained to comprehend the biomedical language they use [35].

In this study, statistically significant differences were observed in the INFLESZ readability score of the different informed consent forms for non-surgical procedures analyzed according to discipline. The complexity of the procedures specific to each specialty may make the drafting of these forms difficult and be a possible cause of this result [28,29,30,31,32,33,36]. In comparison with other research carried out in similar hospital settings in our country, which used the same formal quality criteria for the analysis of the informed consent forms [11,17,27], the existence of a lower number of non-compliances with the formal quality criteria in the forms evaluated has been observed in this study. This result could be a consequence of the hospital’s interest in standardizing the informed consent forms with the participation of all the healthcare professionals involved, including nursing professionals. This interest has made it possible to incorporate in these forms all the information that the patient may need to be able to make his or her decision regarding the procedure to be performed.

As in other studies [11,17,27], the non-compliance with the formal quality criterion in all the informed consent forms for non-surgical procedures analyzed was the verification of the delivery of a copy to the patient. Non-compliance with this criterion prevents a thorough reading of the form and consultation of the details with professionals, family, friends, or even the Internet. This non-compliance hinders adequate comprehension of the information received and the adoption of a decision appropriate to the patient’s needs and interests. The informed consent form read and signed in a short space of time can make the decision too hasty and too conditioned by unresolved doubts that the patient may have, causing the authorization to lack ethical and legal validity [37,38].

Spanish legislation considers that the time spent reading these forms should be inversely proportional to the urgency with which the procedure is to be performed, establishing as a general rule that the patient should have a minimum of 24 h so that he has time to read and adequately comprehend the informed consent form when it is a non-urgent procedure [39].

As in previous studies, other important formal quality criteria that have also been non-compliance in these informed consent forms for non-surgical procedures have been failure to include contraindications (94.59%), alternatives (93.78%), or personalized risks (5.40%) for each procedure [11,17,18,20,27,40,41]. Non-compliance with these formal quality criteria negatively influences the patient’s decision making and legally and ethically invalidates the informed consent process by restricting the patient’s freedom of choice [38].

The statistically significant differences observed between the different informed consent forms of the different disciplines analyzed with respect to compliance with the different formal quality criteria (the name of the procedure appears, the description of the procedure appears, the purpose of the procedure appears, and the alternatives of the procedure appear) and the formal quality score may be due to the characteristics of each of the disciplines or to the specific guidelines established by their respective scientific societies for the preparation of these forms. The unification of the formal quality criteria for all the informed consent forms for the different procedures, regardless of the discipline to which they belong, would allow all these deficiencies to be corrected by increasing the information that these forms provide in order to adapt it to the needs of the patient [11,17,27].

It has been observed that there is no statistically significant correlation between the INFLESZ readability score and the formal quality score of the forms analyzed in this study. This result could be due to the fact that most of the forms evaluated (72.97%) presented a formal quality score of 16 points for non-compliance with the same three formal quality criteria: item 13 (the contraindications of the procedure appear), item 14 (the alternatives to the procedure appear), and item 19 (verification of the delivery of a copy to the patient). If all the forms could be standardized to include these three criteria, the information they can provide would be substantially improved, and patient decision making would be greatly facilitated [11,17,20,27,41].

These deficiencies in readability and formal quality observed in the vast majority of the informed consent forms for non-surgical procedures analyzed make it necessary for nursing professionals to become actively involved in helping to detect and correct these deficiencies, improving their informative capacity and their comprehension by patients. Nursing professionals, due to their direct and continuous work with the patient, are essential to make the biomedical terminology of these forms accessible to the patient. They are also very important in helping to resolve any doubts that the patient may have while reading them and thus facilitate their decision making to authorize the performance of a given procedure [2,23,24,27,42,43].

## 5. Limitations

The main limitation of this study derives from its representativeness, since its external validity could be limited by assessing a local setting. Therefore, further studies are needed to determine whether these results can be generalized to other Spanish hospitals. On the other hand, the analysis of the readability and formal quality of informed consent forms for non-surgical procedures provides limited information that does not guarantee that it can be sufficiently complete and understandable to help patients in their decision making. In order to resolve this aspect, it would be necessary to carry out qualitative research with in-depth interviews that would help to comprehend the real experiences that patients have with this type of form. Nursing professionals, due to the characteristics of their healthcare work, generate an atmosphere of trust with a patient, which makes them very useful for carrying out this type of qualitative research proposed.

## 6. Conclusions

The informed consent forms for non-surgical procedures analyzed presented mostly INFLESZ readability of “somewhat difficult” grade, making them difficult to read and probably to comprehend for a large percentage of patients.

The informed consent forms for non-surgical procedures analyzed showed poor formal quality by not including several important sections such as verification of the delivery of a copy to the patient, contraindications, alternatives, or the personalized risks of each procedure, limiting the information provided to the patient.

The lack of information and the difficulty in comprehension of these informed consent forms for non-surgical procedures pose a major obstacle to the patient’s ability to make a free and voluntary decision about any procedure he is about to undergo.

To ensure that the patient makes a decision that is appropriate to his interests and needs, it is advisable to modify the informed consent forms by including the non-compliance criteria and establishing specific measures to improve their readability and comprehension, fundamentally by adapting them to the cultural level of the specific patients to whom they are addressed.

Due to the importance and complexity of the informed consent process and the characteristics of their healthcare activity, it is necessary for nursing professionals to adopt a relevant and active role in order to improve the readability and formal quality of the informed consent forms, allowing this process to comply with all the legal and ethical objectives entrusted to it.

## Figures and Tables

**Table 1:** INFLESZ scale.

Score	Degree	Level of Education	Type of Publication
<40	Very difficult	University education	Scientific publishing
40–55	Somewhat difficult	Bachelor	Specialized journalism
55–65	Normal	Secondary education	General and sports publications
65–80	Easy enough	Higher primary education	Bestsellers, tabloid journalism and tabloid press
>80	Very easy	Lower primary education	Comics

Note: Adapted from Barrio-Cantalejo et al. [25].

**Table 2:** Formal quality criteria of the informed consent form.

Criteria
1. The hospital name must be shown.
2. The service or unit where the informed consent form is being used must be identified.
3. Spaces must be provided for the informing doctor’s name, last names, registration number, and signature.
4. Spaces must be provided for the first name, last name, identification number, social security number, medical history number, and signature of the patient who will be undergoing the procedure.
5. Spaces must be provided for the name, last name, identification number, and signature of the legal or family representative, or the person who represents the patient.
6. Spaces must be provided for the date and place where the informed consent form was signed.
7. The name of the procedure must be clearly identified.
8. The nature and description of the procedure to be performed must be indicated.
9. The objective of the procedure must be indicated.
10. The relevant or important consequences of the procedure must be shown.
11. The probable risks and the typical risks of the procedure under normal conditions must be indicated.
12. Spaces must be provided for including important personalized risks of the procedure.
13. The contraindications of the procedure must be present.
14. Alternatives to the procedure must be present.
15. The patient’s declaration of having adequately understood the information and having all the doubts clarified must be indicated.
16. The declaration, which states that consent can be revoked at any time, without indicating the cause for this, must be present.
17. A space must be provided for revoking the consent in case the patient considers it necessary.
18. The indication by the patient or the legal representative that the patient provides consent for the procedure must be present.
19. It must be indicated in the document that the patient has been provided with a copy of it.

Note: Source: García-Álvarez et al. [27].

**Table 3:** Readability and formal quality by discipline of informed consent forms for non-surgical procedures.

Discipline	Number of Forms	INFLESZ Score Mean	Formal Quality ScoreMean
All	37	50.76	16.08
Allergy and immunology	1	42.65	13
Cardiology	3	49.75	17
Gastroenterology	10	46.49	16
Hematology	2	51.42	16
Pulmonology	9	54.42	15.89
Neurology	2	45.18	17.5
Radiology	10	53.83	16.10

Note: Created by authors.

**Table 4:** Readability and formal quality of informed consent forms for non-surgical procedures.

Discipline—Form Name	INFLESZ Score	Non-Compliant Criteria	Formal Quality Score
Allergy and immunology—Provocation test	42.65	7, 8, 9, 13, 14, 19	13
Cardiology—Flecainide test	49.63	13, 19	17
Cardiology—Stress test	50.50	13, 19	17
Cardiology—Cardioversion	49.13	13, 19	17
Gastroenterology—Foreign body extraction	47.35	13, 14, 19	16
Gastroenterology—Colonoscopy	46.54	13, 14, 19	16
Gastroenterology—Gastroscopy	49.64	13, 14, 19	16
Gastroenterology—Percutaneous endoscopic gastrostomy	43.80	13, 14, 19	16
Gastroenterology—Gastric polypectomy	45.76	13, 14, 19	16
Gastroenterology—Gastrointestinal bleeding	43.60	13, 14, 19	16
Gastroenterology—Liver biopsy	48.05	13, 14, 19	16
Gastroenterology—Esophageal varices sclerosis	44.83	13, 14, 19	16
Gastroenterology—Paracentesis	48.74	13, 14, 19	16
Gastroenterology—colon polypectomy	46.64	13, 14, 19	16
Hematology—Bone marrow biopsy	54.31	13, 14, 19	16
Hematology—Blood transfusion	48.54	13, 14, 19	16
Pulmonology—Thoracentesis	48.02	13, 14, 19	16
Pulmonology—Non-invasive ventilation	64.94	12, 13, 14, 19	15
Pulmonology—Bronchoscopy	52.07	13, 19	17
Pulmonology—Pleural drainage	55.62	13, 14, 19	16
Pulmonology—Pleural biopsy	62.59	12, 13, 14, 19	15
Pulmonology—Forced spirometry	54.04	13, 14, 19	16
Pulmonology—Walking test	54.86	13, 14, 19	16
Pulmonology—Omalizumab	55.77	13, 14, 19	16
Pulmonology—Nebulization	41.91	13, 14, 19	16
Neurology—Lumbar puncture	50.84	13, 19	17
Neurology—Immunoglobulins	39.52	19	18
Radiology—Cystography	54.63	13, 14, 19	16
Radiology—Opaque enema	55.17	13, 14, 19	16
Radiology—Fine needle biopsy	58.77	13, 14, 19	16
Radiology—Computed axial tomography with contrast	55.07	13, 14, 19	16
Radiology—computed axial tomography	54.67	13, 14, 19	16
Radiology—Punction	46.01	13, 14, 19	16
Radiology—Nephrostomy	55.21	14, 19	17
Radiology—Bowel transit	52.76	13, 14, 19	16
Radiology—Urethrography	51.54	13, 14, 19	16
Radiology—Urography	54.49	13, 14, 19	16

Note: Created by authors.

**Table 5:** Frequencies and percentages of non-compliant formal quality criteria.

Criteria	Frequency	Percentage
7. The name of the procedure appears.	1	2.70%
8. The description of the procedure appears.	1	2.70%
9. The purpose of the procedure appears.	1	2.70%
12. Personalized risks of the procedure appear.	2	5.4%
13. Contraindications to the procedure appear.	35	94.59%
14. Alternatives to the procedure appear.	31	83.78%
19. Verification of delivery of a copy to the patient appears.	37	100%

Note: Created by authors.

**Table 6:** Inferential statistical analysis.

Variables	Statistical Test	Statistical Significance
Discipline and INFLESZ readability score	ANOVA	0.003
Discipline and formal quality criteria	Chi-square	0.000
Discipline and formal quality score	Kruskal–Wallis	0.001
INFLESZ readability score and formal quality score	Spearman	0.349

Note: Created by authors.

## Data Availability

The data used to support the findings of this study are available from the corresponding author upon request.
